# Ageing well with diabetes: the role of technology

**DOI:** 10.1007/s00125-024-06240-2

**Published:** 2024-08-13

**Authors:** Giuseppe Maltese, Sybil A. McAuley, Steven Trawley, Alan J. Sinclair

**Affiliations:** 1 Department of Diabetes and Endocrinology, Epsom & St Helier University Hospitals NHS Trust, Surrey, UK; 2 School of Cardiovascular Medicine & Sciences, King’s College London, London, UK; 3 School of Public Health and Preventive Medicine, Monash University, Melbourne, VIC Australia; 4 Department of Endocrinology & Diabetes, The Alfred, Melbourne, VIC Australia; 5 Department of Endocrinology & Diabetes, St Vincent’s Hospital Melbourne, Melbourne, VIC Australia; 6 Cairnmillar Institute, Melbourne, VIC Australia; 7 Department of Medicine, University of Melbourne, Melbourne, VIC Australia; 8 Foundation for Diabetes Research in Older People (fDROP), Droitwich Spa, UK

**Keywords:** Ageing, Closed loop, Continuous glucose monitoring, Diabetes, Diabetes technology, Frailty, Glucose variability, Hypoglycaemia, Older people, Review

## Abstract

Over the past two decades there has been a substantial rise in the adoption of diabetes therapeutic technology among children, adolescents and younger adults with type 1 diabetes, and its use is now also advocated for older individuals. Older people with diabetes are more prone to experience hypoglycaemia because of numerous predisposing factors and are at higher risk of hypoglycaemic events requiring third-party assistance as well as other adverse sequelae. Hypoglycaemia may also have long-term consequences, including cognitive impairment, frailty and disability. Diabetes in older people is often characterised by marked glucose variability related to age-associated changes such as variable appetite and levels of physical activity, comorbidities and polypharmacotherapy. Preventing hypoglycaemia and mitigating glucose excursions may have considerable positive impacts on physical and cognitive function and general well-being and may even prevent or improve frailty. Technology for older people includes continuous glucose monitoring systems, insulin pumps, automated insulin delivery systems and smart insulin pens. Clinical trials and real-world studies have shown that older people with diabetes benefit from technology in terms of glucose management, reductions in hypoglycaemic events, emergency department attendance and hospital admissions, and improvement in quality of life. However, ageing may bring physical impairments and other challenges that hinder the use of technology. Healthcare professionals should identify older adults with diabetes who may benefit from therapeutic technology and then adopt an individualised approach to education and follow-up for individuals and their caregivers. Future research should explore the impact of diabetes technology on outcomes relevant to older people with diabetes.

**Graphical Abstract:**

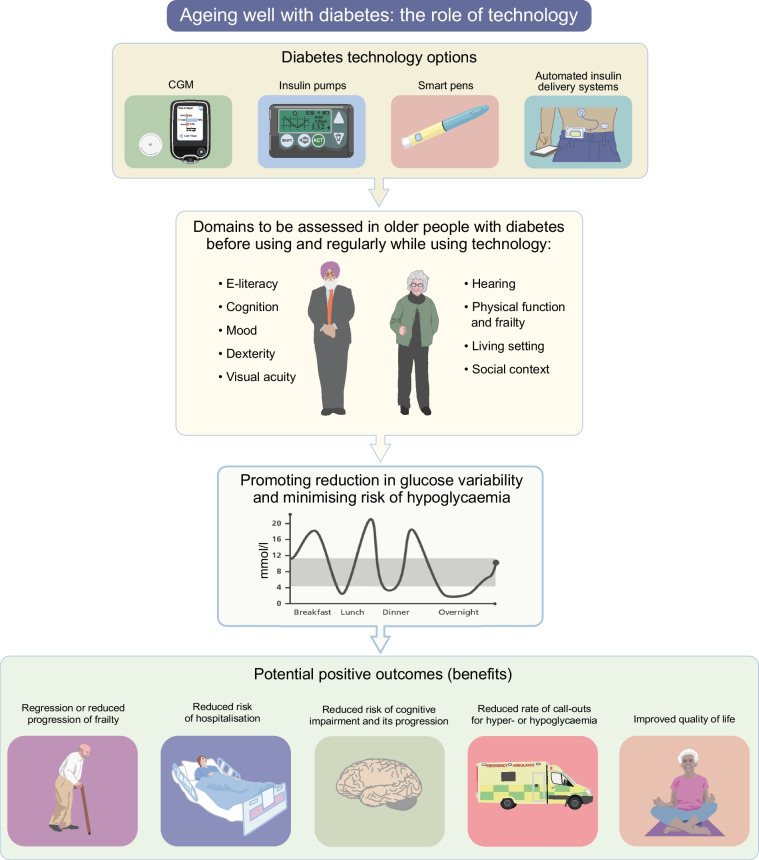

**Supplementary Information:**

The online version contains a slideset of the figures for download available at 10.1007/s00125-024-06240-2.

## Introduction

Diabetes prevalence is increasing worldwide, with global prevalence rates among people aged 65 years and above already exceeding 20%, and with the highest rate (~24%) observed in those aged 75–79 years [1, 2]. In the UK, over a quarter of people with type 1 diabetes and nearly three-quarters of people with type 2 diabetes are aged 60 years and above [3]. The number of older adults with type 1 diabetes is growing progressively due to a reduction in diabetes-related complications and increased life expectancy [4]. Many people with type 1 diabetes now live long healthy lives, even into their eighth decade and beyond [5].

Older adults with diabetes are more susceptible than younger people to recurrent hypoglycaemia and glucose excursions, which can accelerate physical and cognitive decline and increase the risk of adverse events [6]. Technology plays an important role in glucose management and avoidance of hypoglycaemia among older people with diabetes and also appears to improve quality of life and other health-related outcomes [7].

In this article, we review recent evidence on the benefits of available diabetes technology among older people, identify challenges related to its use, describe the assessment process for older people being considered for use of technology and, finally, highlight areas for future research in this field.

## Hypoglycaemia in older people

Diabetes in older people is characterised by marked heterogeneity reflecting factors such as functional status, cognitive status, comorbidities, frailty, duration of diabetes, capacity for self-care, level of family/caregiver support and life expectancy [8]. Given these medical complexities, guidelines for diabetes in older people emphasise that treatment strategies and goals should be individualised following consideration of clinical, psychological, functional and social factors [9].

The ADA recommends glycaemic targets according to an individual’s health status, which can range from ‘healthy’ to ‘complex’ and ‘very complex’ [9]. The ADA also recommends ‘relaxed’ HbA_1c_ targets for older people with a complex or very complex health status, in order to prevent hypoglycaemia [9]. However, HbA_1c_ levels are not associated with hypoglycaemia risk in the older population with diabetes on insulin therapy, and less stringent HbA_1c_ goals do not preclude the risk of hypoglycaemia [10].

As people with diabetes age, they become more prone to hypoglycaemia and their hypoglycaemia awareness may decline, putting them at risk of events requiring third-party assistance. Age per se, through impaired counter-regulatory responses and changes in pharmacokinetics and pharmacodynamics, contributes to the decline in hypoglycaemia awareness [11].

In older people, hypoglycaemia often presents with atypical symptoms, mimicking neurological conditions, or with disorientation or sudden behavioural changes [6] and is one of the most common causes of falls, fractures, cardiac arrythmias and emergency department and hospital attendances [11–13]. Unsurprisingly, the rate of annual hospitalisations due to hypoglycaemia in older people is estimated to be double that for younger individuals and this implies an economic burden for healthcare systems [6].

Recurrent hypoglycaemia is also emerging as a risk factor for geriatric syndromes (particularly cognitive impairment and depression) and appears to be associated with significant morbidity, frailty and disability [6]. The relationship between hypoglycaemia and cognitive impairment is well established and bidirectional. In turn, cognitive impairment in older people with diabetes is commonly associated with impaired awareness of hypoglycaemia and glucose variability. In a population-based prospective cohort study of 783 older people with diabetes, over the 12 year follow-up period, individuals who experienced a severe hypoglycaemia event were twice as likely to develop dementia, and individuals who developed dementia were three times more likely to have a subsequent severe hypoglycaemia event [12].

Hypoglycaemia also affects the quality of life of older people. Individuals who experience frequent hypoglycaemia or hypoglycaemia fear tend to avoid activities such as physical exercise and this worsens the feeling of anxiety and leads to social isolation [13]. Minimising the risk of and fear of hypoglycaemia is therefore a key aim in the management of diabetes in older people, in order to improve health-related outcomes and quality of life. However, relaxing HbA_1c_ goals does not preclude the risk of hypoglycaemia and may worsen outcomes for older people with diabetes.

## Glucose variability and risk of adverse clinical events

For decades, HbA_1c_ has been the gold standard parameter for assessing glycaemic management in people with diabetes; however, it does not take into consideration excursions in blood glucose levels known as glucose variability, and in the older population may not reflect mean glucose levels equivalent to those for younger adults because of comorbidities that affect the lifespan of red blood cells [14, 15]. Thus, there are major shortcomings with using HbA_1c_ as the sole parameter for measuring glycaemic goals in older people.

Glucose variability is receiving particular attention as a parameter for assessing glycaemic management as it can increase both the risk of hypoglycaemia and fluctuations in the hyperglycaemia range [16]. Several studies have shown that glucose variability has more deleterious effects than sustained hyperglycaemia in the development of diabetes-related complications and is associated with an increased risk of mortality [17, 18]. It is thought that glucose variability accelerates the ageing process and the progression of CVD through enhanced oxidative stress, low-grade inflammation and endothelial dysfunction [19, 20]. Attenuated glucose variability in the absence of diabetes has been linked to healthy ageing and longevity [21]. Compared with young adults with diabetes, older people with diabetes appear to have a higher degree of glucose variability, which predicts severe and nocturnal hypoglycaemia [22].

There are two types of glucose variability: (1) long-term glucose variability, based on serial measurements over a long period of time and involving HbA_1c_, serial fasting plasma and postprandial glucose measurements; and (2) short-term glucose variability, represented by within-day and between-day glucose variability [23]. The most popular metrics for glucose variability are the % CV, which is correlated with risk of hypoglycaemia, and the SD, which is highly correlated with most measures of glucose variability, including mean amplitude of glycaemic excursion, IQR, mean of daily differences and average daily risk range [23].

Table 1 describes the main studies that have examined the relationship between glucose variability and adverse events in older people with diabetes. In a study including 130 adults with type 1 diabetes aged ≥65 years, those with high glucose variability (>36% CV) spent more time in hypoglycaemia (both <3.9 and <3.0 mmol/l) than those with low glucose variability (≤36% CV; 84 vs 31 min/day, *p*<0.0001, and 46 vs 8 min/day, *p*<0.001, respectively). Importantly, when HbA_1c_ was higher than the glucose management indicator, hypoglycaemia of longer duration was observed. % CV and the glucose management indicator are therefore better indicators for risk of hypoglycaemia than HbA_1c_ [24].

**Table 1:** Key studies exploring the relationship between glucose variability and adverse events in older people with diabetes

Study	Objectives	Method used to assess GV	Type of diabetes	Sample size	Age (years)^a^	Main findings
Single-centre study, Toschi et al 2020 [24]	Evaluate relationship between GV and risk of hypoglycaemia	CGM	T1D	130	71 ± 5	Participants with high GV (>36% CV) spent more time in hypoglycaemia (<3.9 mmol/l and <3.0 mmol/l) than those with low GV (≤36% CV; 84 min/day vs 31 min/day, *p*<0.0001, and 46 min/day vs 8 min/day, *p*<0.001, respectively)
Population-based retrospective cohort study, Li et al 2017 [26]	Evaluate relationship between visit-to-visit variations in FPG and HbA_1c_ and AD	Variations in FPG and HbA_1c_ represented by the CV	T2D	16,706	69 ± 5.9	FPG CV and HbA_1c_ CV were predictors of AD, with HRs of 1.27 (95% CI 1.06, 1.52) for the third tertile in FPG CV and 1.32 (95% CI 1.11, 1.58) for the third tertile in HbA_1c_ CV
Observational study, Chung et al 2021 [27]	Investigate relationship between GV and frailty	CGM	T2D	48	Healthy/pre-frail: 78.5 ± 7.4; frail: 80.8 ± 6.5	No statistically significant differences in TIR (60.0% vs 44.2%, *p*=0.053), TAR (39.2% vs 55.0%, *p*=0.053), % CV and TBR (*p*>0.1) between healthy/pre-frail and frail individuals. CGM metrics (GMI-HbA_1c_, TAR) were higher in the frail group only in the postprandial periods (all *p*<0.05)
Multicentre, prospective, observational cohort study, Fung et al 2021 [28]	Investigate relationship between frailty, dysglycaemia and mortality risk	CGM	T2D	215	Median (IQR) 74 (71–78)	Decreased TIR and increased TAR metrics were strongly correlated with increased frailty and hyperglycaemia (*p*<0.00001), whereas TBR metrics (<3.9 and 3.0–3.8 mmol/l) were marginally (*p*<0.05) or not different (<3.0 mmol/l, *p*=0.18) between frailty levels
Data analysis from three prospective studies, Idrees et al 2023 [29]	Investigate effects of frailty on glucose control among hospitalised individuals	CGM	T2D	263	103 aged ≥60 years; 160 aged <60 years	Higher degree of frailty was associated with higher % CGM <3.9 mmol/l and <3.0 mmol/l
Retrospective cohort study, Forbes et al 2018 [30]	Investigate association between HbA_1c_ variability over time and mortality risk	Score based on HbA_1c_ variability over time	T1D and T2D	54,803	Women: 79 ± 6.1; men: 77.5 ± 5.4	Survival rate was inversely associated with GV
Observational study, Ma et al 2023 [31]	Explore relationship between blood glucose fluctuations and sarcopenia	CGM	T2D	280 (sarcopenic: *n*=43; non-sarcopenic: *n*=237)	≥60	Prevalence of sarcopenia was inversely associated with TIR values (40.48% for TIR≤50%, 20.41% for 50%<TIR≤70%, 8.47% for TIR>70%). No difference in GV parameters (CV, MAGE) between the two groups (CV: *p*=0.872; MAGE: *p*=0.366)
Observational study, Zhang et al 2021 [32]	Investigate effect of GV on risk of arrythmias in older and middle-aged people	CGM	T2D	107 (older: *n*=73; middle-aged: *n*=34)	Older: 81.1 ± 5.3; middle-aged: 56.7 ± 5.2	Older people had greater GV (*p*<0.05) and were more prone to supraventricular and ventricular arrhythmias than younger individuals (*p*<0.005)

^a^Data are mean ± SD unless indicated otherwise

AD, Alzheimer’s disease; CGM, continuous glucose monitoring; FPG, fasting plasma glucose; GMI, glucose management indicator; GV, glucose variability; MAGE, mean amplitude of glycaemic excursions; T1D, type 1 diabetes; T2D, type 2 diabetes; TAR, time above range; TBR, time below range; TIR, time in range

There is a paucity of data on the effect of glucose variability on physical and cognitive function and frailty. Among people with type 2 diabetes, high glucose variability has been associated with cognitive impairment [25]. In the Taiwan diabetes study, visit-to-visit variations in fasting plasma glucose levels and HbA_1c_ have been associated with Alzheimer’s disease [26]. Several studies have investigated the relationship between glucose variability and frailty. Chung et al divided 48 individuals with type 2 diabetes aged ≥65 years and using continuous glucose monitoring (CGM) into two groups: ‘healthy and pre-frail’ (*n*=24) and ‘frail’ (*n*=24). CGM was used for 6.9 days and the authors found higher CGM metrics (particularly post-lunch time above range) in the frail group (*p*<0.05) [27]. These findings may not reflect glucose variability per se as there were no differences between groups in % CV/mean amplitude of glycaemic excursion measurements. A study by Fung et al [28] showed a relationship between incremental frailty and increased dysglycaemia in older adults with type 2 diabetes on insulin, whereas Idrees et al demonstrated an association between frailty and hypoglycaemia in non-acute settings [29].

In older people, glucose variability can influence mortality risk. In a 5 year retrospective cohort study including data from 54,803 individuals aged ≥70 years with type 1 or type 2 diabetes, Forbes et al evaluated the effect of glucose variability on mortality risk by using a new metric for glucose variability and found that mortality risk increased with increasing glycaemic variability [30]. Ma et al explored the relationship between sarcopenia and glucose variability in a study including 280 individuals with type 2 diabetes aged ≥60 years who were divided into a sarcopenic group and a non-sarcopenic group and wore a continuous glucose monitor for 3 days [31]. The authors found lower prevalence of sarcopenia with higher time in range (TIR) values (40.48% for TIR≤50%, 20.41% for 50%<TIR≤70%, 8.47% for TIR>70%). However, parameters of glucose variability (CV, mean amplitude of glycaemic excursions) were not different between the two groups (CV *p*=0.872, mean amplitude of glycaemic excursions *p*=0.366) [31].

In another study, Zhang et al demonstrated that older people, when compared with middle-aged individuals, have a greater glucose variability which is associated with an increased risk of supraventricular and ventricular arrythmias [32]; these studies are described in Table 1.

Glucose variability can also impact individuals’ psychological well-being, whereas its attenuation can lead to improvements in quality of life [33].

In conclusion, the literature indicates that wide glucose variability is associated with negative health-related outcomes in older people with diabetes and its attenuation might positively impact quality of life and increase life expectancy.

## Diabetes technology in older people

A key aim of current management approaches to diabetes in older people is to support them to achieve healthy glucose levels and minimise the risk of hypoglycaemia.

The use of technology by older people with diabetes is growing and has been shown to have a remarkable impact on their diabetes-related outcomes and quality of life. However, technology use comes with associated challenges that need to be addressed.

The use of diabetes technology in older adults is discussed in the following sections; under the umbrella term ‘diabetes technology’ we have included connected insulin pens, CGM, insulin pumps and automated insulin delivery (AID) systems.

### Continuous glucose monitoring

Finger-prick blood glucose monitoring has long been a key component of the self-management of people living with diabetes. However, blood glucose monitoring provides a static view of the glucose profile and does not measure glucose excursions or undetected hypoglycaemia. Attenuating glucose variability and the risk of hypoglycaemia are major goals of diabetes management in older people. CGM, both real-time and ‘flash’ (the latter requiring manual swiping and now relatively obsolete), provides a dynamic view of glucose patterns, which is an advantage in older people at increased risk of hypoglycaemia. Many devices can also predict low and high glucose levels and give predictive warning alerts.

Most CGM devices have a ‘share’ feature that allows healthcare professionals, relatives and caregivers to review data remotely. Remote monitoring by healthcare and/or family members is a unique advantage of CGM in older people with diabetes and may be of particular value for those individuals living alone and for whom extra assistance is required. Diabetes technology also has the potential for seamless integration with other wearable devices aimed at tracking vital parameters. For example, the Dexcom and Freestyle Libre CGM systems allow users to share their glucose readings and alarms with up to 10 or 20 people, respectively. However, it is important to highlight that, although this feature can be valuable, some older adults may prefer not to share detailed insights, even with close family members such as spouses; this preference may vary depending on their level of comfort and degree of disability. Therefore, the decision to use the ‘share’ feature should be personalised according to the specific needs and preferences of each individual. However, there is evidence to suggest that older adults do not mind using the share feature with their caregivers [34].

**Fig. 1:**
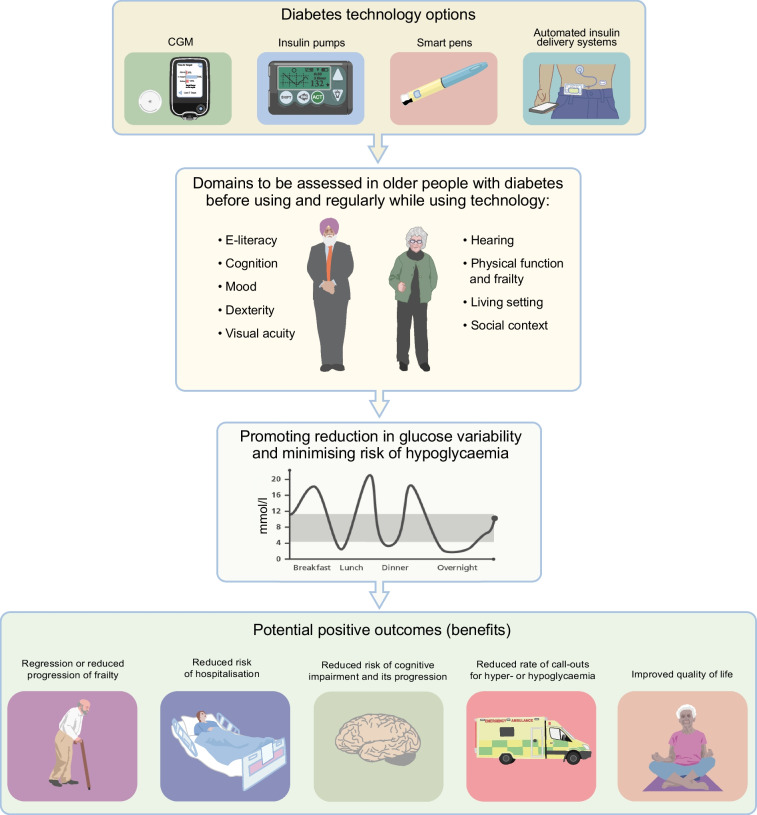
Benefits of technology in older people with diabetes. This figure is available as part of a downloadable slideset


Figure 1 illustrates some of the potential key benefits of CGM in older people with diabetes, and the text box ‘Case vignette: improved glucose levels and quality of life after escalation of diabetes technology’ provides an example of an individual who showed a profound improvement in quality of life with hypoglycaemia reduction after commencement of CGM as part of a clinical trial [35]. CGM has been shown to improve TIR and reduce time below range (TBR), and the risk of hypoglycaemia and hospitalisations [36]. CGM also has a positive impact on psychological outcomes, patient satisfaction and quality of life [36]. The Endocrine Society and the American Association of Clinical Endocrinology (AACE) recommend the use of CGM for people aged ≥65 years on insulin, even if they have physical and cognitive limitations [37, 38]. Consistent with these recommendations, the use of CGM in older people with type 1 and type 2 diabetes is gradually growing, as reported in a recent study from Germany using data from the Diabetes Prospective Follow-up Registry [39]. The benefits of technology shown in Fig. 1 also apply to pump therapy and include mitigation of glucose variability and reduction in hypoglycaemia risk. Clearly, there are many overlapping benefits between CGM and pump therapy in individuals with diabetes.

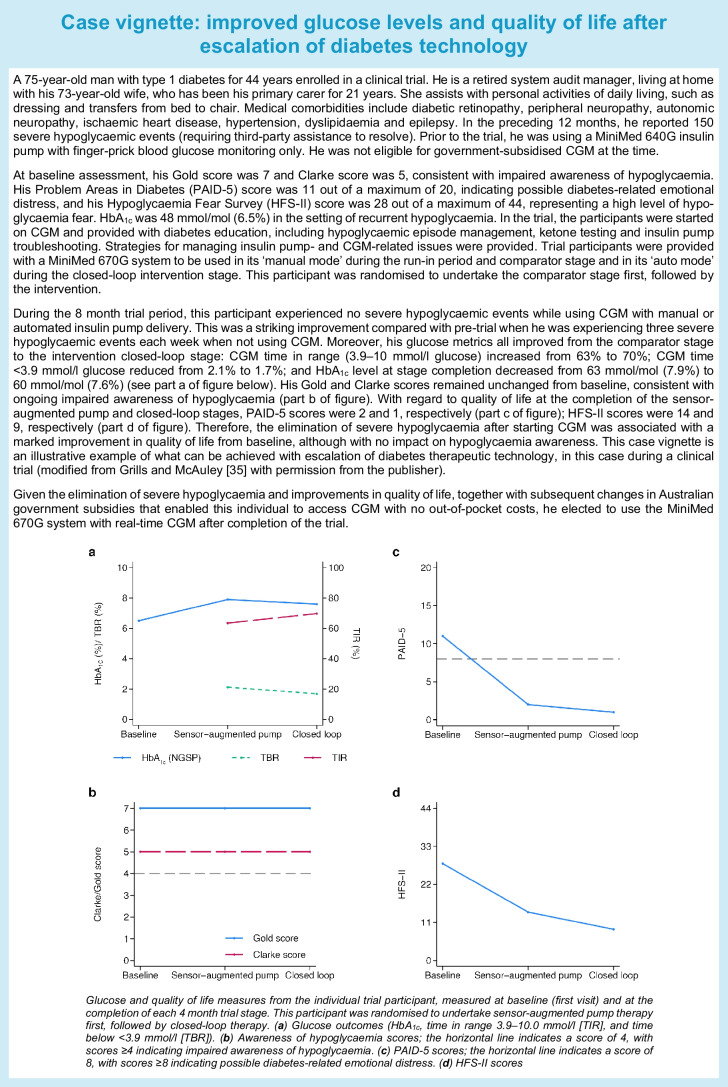


To date, the most robust evidence on the benefits of CGM for older adults derives from a few retrospective, qualitative and survey studies and RCTs, including a subanalysis of the DIAMOND study (Table 2). The DIAMOND study was a large RCT involving people with type 1 diabetes (aged 26–79 years) treated with insulin multiple daily injections that compared CGM with blood glucose monitoring [40]. This trial found that CGM was associated with greater reduction in HbA_1c_ than blood glucose monitoring [40]. The seven studies included in Table 2 [41–47] found benefits of CGM in older people with diabetes, including reductions in HbA_1c_, less time above range, fewer episodes of hypoglycaemia or diabetic ketoacidosis, high levels of satisfaction, less TBR and more TIR.

**Table 2:** Key studies investigating CGM in older people with diabetes

Study	Type of diabetes	Sample size	Age (years)^a^	Comparison	Follow-up (weeks)	Main outcomes
Subanalysis of DIAMOND, Ruedy et al 2017 [41]	T1D and T2D	116	67 ± 5	CGM vs BGM	24	Greater reductions in HbA_1c_ (−9.8 ± 7.7 mmol/mol [−0.9 ± 0.7%] vs −5.5 ± 7.7 mmol/mol [−0.5 ± 0.7%], respectively [adjusted difference in mean change −4.4 ± 1.1 mmol/mol [−0.4 ± 0.1%], *p*<0.001]) and TAR (*p*=0.006) and lower GV (*p*=0.02) in the CGM group vs BGM group
WISDM, Pratley et al 2020 [42]	T1D	203	Median (IQR) 68 (65–71)	CGM vs BGM	24	Lower TBR in the CGM group vs BGM group (2.7% vs 4.9%; adjusted treatment difference −1.9%)
Subanalysis of MOBILE, Bao et al 2022 [43]	T2D	175	≥65 (range 65–79)	CGM vs BGM	32	Greater reduction in HbA_1c_ in the CGM group vs BGM group (mean change −11.8 mmol/mol [−1.08%] vs −4.2 mmol/mol [−0.38%] [adjusted mean difference −0.65%, 95% CI −1.49, 0.19]). For TIR, mean adjusted treatment group difference was 19% (95% CI 4, 35, *p*=0.01)
Retrospective study, Guerci et al 2023 [44]	T2D	38,312	≥65	FM	96	Reduction in adverse diabetes events (−34% and −40% after 12 and 24 months’ use of FM, respectively). For those aged 70–79 and ≥80 years, significant reductions in SH 24 months following FM initiation (–30% and –46%, respectively)
Retrospective observational cohort study, Reaven et al 2023 [45]	T1D and T2D	20,721	66.7 ± 9.8	CGM vs BGM	48	Significantly greater improvement in HbA_1c_ in both T1D (−2.8 mmol/mol [−0.26%], 95% CI −0.33, −0.19) and T2D (−3.8 mmol/mol [−0.35%], 95% CI −0.40, −0.31) in CGM group vs non-users. In those with T1D, significantly lower risk of hypoglycaemia (HR 0.69, 95% CI 0.48, 0.98) and all-cause hospitalisation (HR 0.89, 95% CI 0.83, 0.97) in CGM group vs non-users
Survey study, Polonsky et al 2016 [46]	T1D and T2D	285	70.7 ± 5.0	CGM vs BGM	24	Users reported fewer episodes of SH than non-users for the previous 6 months (*p*<0.01) and greater reductions in emergency department attendance and paramedic-led home visits (*p*<0.01), better general well-being (*p*<0.001) and less distress and hypoglycaemia fear (*p*<0.05)
Qualitative study, Litchman et al 2017 [47]	T1D	22	70 ± 4.7	CGM users vs CGM non-users	–	CGM users less likely than non-users to experience SH (*p*=0.02) or hypoglycaemia resulting in a fall or the inability to drive a motor vehicle (*p*=0.01)

^a^Data are mean ± SD unless indicated otherwise

BGM, blood glucose monitoring; FM, flash monitoring; SH, severe hypoglycaemia; T1D, type 1 diabetes; T2D, type 2 diabetes; TAR, time above range; TBR, time below range

CGM therefore has demonstrated benefits in terms of glycaemic outcomes, but there is still concern about its feasibility of use, as some technical aspects of CGM devices may impact older people’s confidence in their ability to manage them. Only one small study has demonstrated that the use of CGM is associated with high participant satisfaction and device acceptability and no additional distress [48].

### CGM metric targets

In a consensus published in 2019, Battelino et al provide CGM targets for all older adults as well as people at high risk of severe hypoglycaemia due to duration of diabetes, duration of insulin treatment and impaired awareness of hypoglycaemia [49]. For this ‘high-risk’ group, the authors recommend a less stringent TIR (3.9–10.0 mmol/l glucose) target of >50% (vs >70% in general recommendations) and a TBR (<3.9 mmol/l glucose) target of <1% (vs <4% in general recommendations) [49], irrespective of frailty and functional status. Consensus CGM metric targets for older people with diabetes that take into consideration important factors such as comorbidities, health status and presence and severity of frailty are also needed. Trawley et al compared CGM data from older adults with type 1 diabetes collected during the run-in phase of the ORACL trial with the consensus-recommended CGM targets for all older adults with diabetes [50]. The authors highlighted that the recommended uniform TIR of >50% for all older adults (equating to an HbA_1c_ of 67 mmol/mol [8.3%]) is inappropriately low for non-frail older adults using real-time CGM technology [50]. CGM targets should be revisited and personalised based on clinical aspects such as frailty, functional status and comorbidities, as well as the use or non-use of CGM or AID systems [50].

### Bluetooth-enabled pens

In older people with diabetes, insulin dose errors, including missed insulin or double doses, are common and may account for suboptimal glycaemic management or unexplained glucose variability [51]. Advising insulin dose changes based on an incorrect assumption that an individual is following the prescribed insulin treatment can potentially lead to overtreatment and hypoglycaemia.

Bluetooth-enabled insulin pens, also known as smart pens, have been introduced to record insulin doses and time of administration. These pens can be extremely useful for older people who have mild memory impairment but who are otherwise capable of self-managing their diabetes (e.g. for reducing risk of hypoglycaemia and improving insulin treatment adherence). The concomitant use of smart pens and CGM has the potential to improve adherence to intended insulin dosing, detect accidental incorrect dosing and reduce the risk of problematic hypoglycaemia [52]. A downside of these pens is the need to replace the insulin cartridge, which may be a complex task for individuals with impaired dexterity without caregiver assistance.

Available smart pens can record and store data about insulin doses and injection timing and produce downloadable reports [53]. Through a ‘share’ function, caregivers and relatives can monitor treatment adherence. Smart pens also represent an excellent option for care home residents who are able to self-manage their diabetes under supervision. However, despite the proven benefits of smart pens in terms of TIR improvement and reduction in hypoglycaemia frequency, they have not yet been fully implemented in clinical practice [52].

### Insulin pumps and AID systems

Insulin pump therapy can be a viable option for selected older adults who meet the clinical criteria. Indications for insulin pump therapy for older adults are the same as those for younger adults [54]. Although there is a large body of evidence demonstrating the efficacy of insulin pump therapy among younger adults with type 1 diabetes [54], evidence among older people is limited [55, 56]. A small number of studies and retrospective analyses have shown that insulin pump therapy is safe and effective in improving glycaemic management and reducing the risk of hypoglycaemia among adults with type 1 diabetes aged >60 years, but there are very minimal data from older groups and from individuals with medical complexity and physical and cognitive impairments. Clinicians must be mindful of comorbidities, and their treatments, which can contribute to type 1 diabetes management complexity, such as emergency management when insulin pump delivery interruption occurs [57].

Insulin pump therapy for older people is associated with benefits beyond glycaemic management and hypoglycaemia and has a positive impact on daily life (Table 3) [58–63]. Insulin pump therapy can also reduce the risk of hypoglycaemia and hospitalisation in both younger and older people, as demonstrated by a retrospective study in a Polish population [64].

**Table 3:** Key studies investigating insulin pump therapy and AID systems in older people with diabetes

Study	Type of diabetes	Sample size	Age (years)^a^	Comparison	Follow-up (weeks)	Main outcomes
Prospective, observational, single-centre study, Pintaudi et al 2023 [58]	T1D	18	74.1 ± 7.1	HCL system (MiniMed 780G)	48	HCL system was associated with a significant improvement in HbA_1c_ (mean ± SD 59.9 ± 10.5 mmol/mol [7.6% ± 3.1%] at baseline vs 53.2 ± 6.0 mmol/mol [7.0% ± 2.7%] at 1 year, *p*=0.01; mean difference 6.8 ± 10.3 mmol/mol [2.8% ± 3.1%]) and increase in TIR at 48 weeks (*p*<0.0001)
Open-label, randomised crossover trial (ORACL), McAuley et al 2022 [59]	T1D	30	67 ± 5	HCL system (MiniMed 670G) vs SAP	16	Mean (SD) TIR was higher in the HCL group than SAP group (75.2% [6.3] vs 69.0% [9.1], respectively; difference 6.2 percentage points [95% CI 4.4, 8.0]; *p*<0.0001) and the HCL group had a lower time in hypoglycaemia (<3.9 mmol/l) by a median of 0.5 percentage points (95% CI 0.3, 1.1; *p*=0.0005) vs SAP therapy
Retrospective analysis of electronic health records, Toschi et al 2022 [60]	T1D	48	70 ± 4	HCL system (Control-IQ)	12	CGM metrics showed an increase in mean ± SD TIR (from 62% ± 13% to 76% ± 9%; *p*<0.001) and a reduction in median (IQR) TBR (<3.9 mmol/l; from 2% [1–3%] to 1% [1–2%]; *p*=0.03) and mean ± SD TAR (>10.0 mmol/l; from 30% ± 11% to 20% ± 9%; *p*<0.001) at 3 months
Cross-sectional survey, Chakrabarti et al 2022 [61]	T1D	30	69 ± 5	–	–	Insulin pump therapy was associated with high levels of self-confidence in managing diabetes around exercise
Multinational, randomised, open-label crossover trial, Boughton et al 2022 [62]	T1D	37	Median [IQR] 68 [63–70]	HCL system (CamAPS FX) vs SAP	16	HCL system was associated with an improvement in TIR of 8.6 percentage points vs SAP through a reduction in time spent with glucose levels >16.7 mmol/l. There were no differences in TBR (<3.9 mmol/l) between the two groups
Post hoc analysis of a RCT, Thabit et al 2023 [63]	T1D	37	Median [IQR] 68 [63–70]	HCL system (CamAPS FX) vs SAP	16	There were no significant differences in sleep traits between the HCL and SAP groups

^a^Data are mean ± SD unless indicated otherwise

HCL, hybrid closed-loop; SAP, sensor-augmented pump; T1D, type 1 diabetes; T2D, type 2 diabetes; TAR, time above range; TBR, time below range; TIR, time in range

The body of evidence on the use and benefits of diabetes technology is even more limited for AID systems (Table 3). Currently available AID systems, also referred to as hybrid closed-loop therapy, include an insulin pump and linked CGM device. The CGM measures interstitial glucose levels and an algorithm housed in the pump or in a mobile app adjusts the amount of insulin delivered according to the glucose levels. These current AID systems are considered to be ‘hybrid’ systems, as some information from the user (e.g. meal carbohydrate content) is still required to be entered manually.

The limited literature on the advantages of AID in older people suggests the need for further work. In a small study including 18 older adults with type 1 diabetes (mean ± SD age 74.1 ± 7.1 years) conducted by Pintaudi et al [58], the use of AID resulted in improvements in HbA_1c_ at 1 year (mean ± SD 60 ± 11 mmol/mol [7.6% ± 3.1%] at baseline vs 53.2 ± 6.0 mmol/mol [7.0% ± 2.7%] at 1 year, *p*=0.01; mean difference 7 ± 10 mmol/mol [2.8% ± 3.1%]) and TIR at 48 weeks (*p*<0.0001) compared with baseline.

Two key randomised AID trials including individuals with type 1 diabetes aged >60 years have been recently published. The ORACL trial, a two-centre Australian study including 30 individuals with type 1 diabetes aged >60 years, compared a first-generation AID system (MiniMed 670G) with SAP therapy [59]. Compared with SAP therapy, using an AID system resulted in reduction in time spent in hypoglycaemia (<3.9 mmol/l) by a median of 0.5 percentage points (95% CI 0.3, 1.1; *p*=0.0005) [59]. There was a threefold reduction in nocturnal hypoglycaemia with AID and this effect was still detectable during actigraphy-measured sleep periods [65]. It is worth highlighting that in the trial only 20% of participants had mild cognitive impairment (none had moderate or severe cognitive impairment) and one-third had impaired awareness of hypoglycaemia. Only 20% were pre-frail and none was frail [59].

In a similar multinational, randomised, open-label crossover study conducted by Boughton et al [62], 37 people with type 1 diabetes aged ≥60 years (median [IQR] age 68 [63–70] years) were randomised to either an AID device (CamAPS FX) or SAP therapy for a period of 16 weeks. AID improved TIR by 8.6% through a reduction in time spent with glucose levels >16.7 mmol/l. There were no differences in time spent in hypoglycaemia (<3.9 mmol/l) between the two treatments [62]. Possible limitations of this trial were enrolling participants who were not necessarily representative of the heterogeneity of older people with type 1 diabetes and not screening for impairments in dexterity, vision, hearing or cognition. A trial subanalysis explored patient-reported outcomes [66]. Findings included significant improvements in diabetes distress overall (and with improvements in the powerlessness and physician distress subscales), in addition to improvement in trust in glucose monitoring [66].

A few additional small studies and RCTs have also shown benefits of insulin pump therapy in older people with type 2 diabetes [67, 68].

In young adults with type 1 diabetes the use of AID is associated with quality of life benefits, including a reduction in diabetes-related psychological burden and an improvement in sleep [69]. This would be an important advantage in older people as they tend to have disrupted sleep because of hyper- or hypoglycaemia and fear of hypoglycaemia. However, to date, the findings on the effects of AID on sleep quality have been disappointing. In the ORACL trial, sleep quality recorded daily was worse in those participants using the first-generation AID system (*p*=0.006) [65]. Similarly, in a post hoc analysis of the RCT conducted by Boughton et al [62], the use of the CamAPS FX device did not produce any improvement in sleep traits [63]. Lack of effect of an AID (vs SAP) on sleep parameters was also reported by Bisio et al in a small pilot trial [70].

Current-generation AID systems, with CGM no longer requiring calibration and algorithms with greater automation than in earlier systems, can now be used effectively in clinical care, even for older individuals with extensive comorbidities and suboptimal system interactions, such as missing meal announcements. However, to date, AID clinical trials have largely excluded such individuals.

## Challenges related to the use of technology in older people

**Fig. 2:**
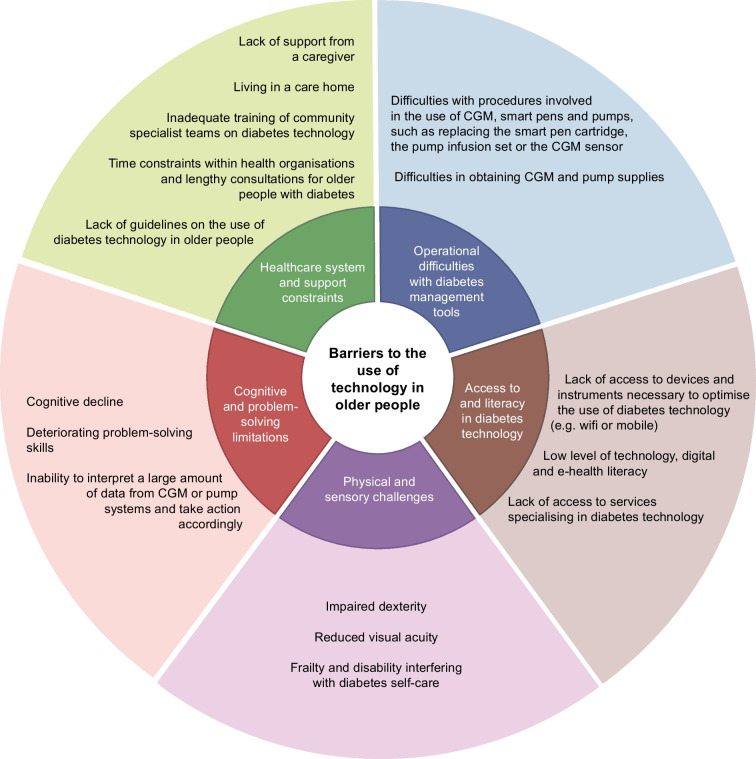
Key barriers to the use of technology in older people. This figure is available as part of a downloadable slideset

The use of technology holds promise to improve multiple outcomes in older people with diabetes, but its implementation is accompanied by many barriers that need to be overcome [71]. Figure 2 lists the key barriers to the use of technology in older people with diabetes.

Ageing is commonly associated with cognitive and physical decline and dexterity and visual impairments that interfere with diabetes self-care and do not allow the effective and safe use of technology devices unless the person with diabetes is supported by a relative or caregiver [71]. In a retrospective analysis by Toschi et al demonstrating the benefits of AID in older adults in terms of TIR and TBR, physical issues (dexterity and visual problems) and cognitive challenges were barriers to use for a few participants [60].

The text box ‘Case vignette: comorbidities and cognitive decline limiting the use of diabetes therapeutic technology’ provides an example where an individual’s comorbidities, cognitive decline and social situation were no longer conducive to the safe use of diabetes technology.

Personal preference and living circumstances are also important considerations. Some older individuals with diabetes are more inclined than others to use technology, and the level of available support, such as from a carer or family member, and/or proximity to specialised/tertiary centres where medical staff have expertise in the field of technology are relevant factors. Frail, non-independent individuals may need to live in care homes, where staff are less likely to possess the necessary skills to manage CGM devices and pumps. All of these factors can potentially result in age-related inequities.


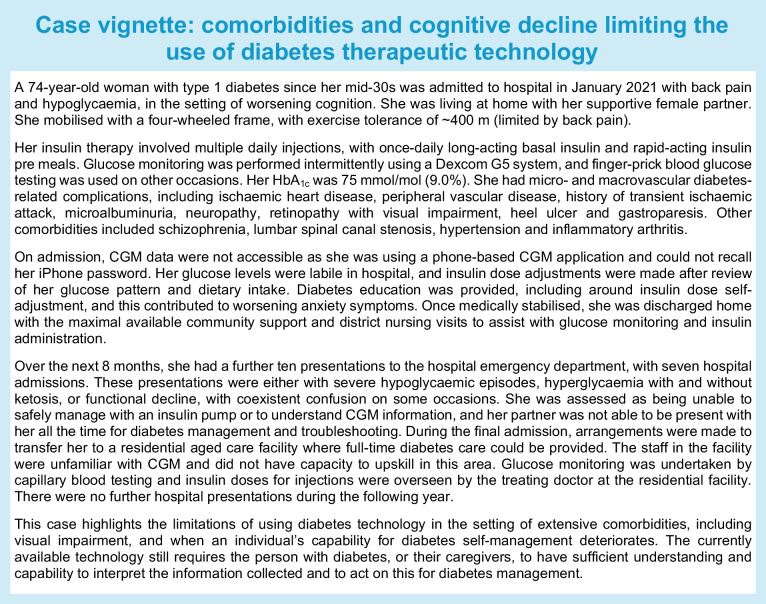


Structural discrimination may also have a negative impact on access to technology [71]. Some groups of older people from ethnic minorities or with low incomes, limited education and low levels of health literacy may be socially marginalised and face barriers to internet and broadband access. Tackling these structural inequities necessitates reforms in both physical and social environments to promote equality across populations [71].

Among older adults, there can also be substantial variation in technology and digital literacy [7]. Technology literacy is defined as the ability to use technology devices safely and can be potentially enhanced by targeted education [7]. Digital literacy is defined as the ability to locate and consume, create and communicate digital content [7]. Older individuals may also have variable e-health literacy, a characteristic bridging health and technology [7]. As explained by Norman and Skinner, e-health literacy is the ‘ability to seek, find, understand, and appraise health information from electronic sources and apply the knowledge gained to addressing and solving a health problem’ [72].

CGM devices provide a large amount of data requiring interpretation and numeracy skills. Regardless of the level of literacy, data can be overwhelming for an older person with cognitive impairment and can trigger anxiety leading to rushed actions, insulin dosing errors and consequent hypoglycaemia.

Some procedures involved in the use of technology may represent complex tasks for older people. As a result of these difficulties, for instance, the infusion set may be left in place for longer than recommended, or the infusion set items may be reused or the pump discontinued. In addition, there is concern that older people may not be able to deal with issues related to pump function or, in the worst-case scenario, with pump failure, as problem-solving skills are known to decline with ageing.

There also challenges related to time constraints within health organisations. Because of cognitive impairment and medical complexity, the review of an older person and training of a family member or caregiver on the use of technology may require lengthy consultations and frequent follow-up. Another aspect to consider is the training of healthcare professionals on the use of technology. Older people with diabetes, particularly those who are frail, are often under the care of community diabetes specialist services. In this setting, clinicians are not always proficient in using CGM and other technology devices. Furthermore, diabetes specialists may not be familiar with tools for assessing cognition, mood, physical function and frailty and this raises the question whether older people with diabetes should be managed within dedicated multidisciplinary clinics.

Because of these barriers to the use of technology by older people, despite the advantages of CGM devices and pumps, most healthcare professionals prefer to continue with multiple daily injections. The lack of guidance on how to support older people with declining self-care skills in the use of technology may further amplify this attitude.

## Assessment of older people for use of diabetes technology

Diabetes self-management requires lifelong commitment to numerous highly complex tasks including glucose monitoring, insulin dose adjustments and administration, hypoglycaemia management, self-coordination and planning. All of these tasks, requiring problem-solving and cognitive skills, are essential to achieve successful health-related outcomes.

Ageing and diabetes both increase the risk of comorbidities, including cognitive impairment, depression, visual acuity reduction and impaired dexterity, which can potentially cause physical and mental decline and frailty and interfere with individuals’ self-care abilities [73]. A list of domains to be assessed in older people before and while using technology is provided in Fig. 1, and some key assessments [74–76] are described in Table 4, which also includes key outcome measures relating to the potential positive impact of technology in older people with diabetes.

**Table 4:** Domains to assess in an older person with diabetes before and during the use of diabetes technology

Domain	Importance	Impact of ageing and comorbidities	Recommended assessments	Technology impact and recommendations
Glucose monitoring, insulin dosing (adjustments/administration), hypoglycaemia management	Essential for overall health	Increased risk of cognitive impairment, depression, dexterity and visual acuity issues	Cognitive screening, mood assessment, dexterity assessment and visual acuity checks	Appropriately selected devices can enhance outcomes; need for user-friendly designs for older adults with diabetes
Cognitive skills (memory, learning and executive functioning)	Essential for ability to self-care	Higher incidence of cognitive impairment with age [73]	Annual cognitive impairment screening (e.g. Mini-Cog) [75]	Supporting independent use of CGM and insulin pump therapy such as interpretation of data, responding to device alerts and liaison with health professionals
Mood	Affects ability to self-care [73]	Depression associated with decline in cognitive function	Annual screening for depression (Geriatric Depression Score [GDS]) [76]	Early detection of mood disturbance can enable effective treatment to commence, thereby enhancing diabetes self-care abilities
Dexterity	Necessary to operate specific devices	Impairment due to age-/diabetes-related pathologies [73]	Hand function and motor performance	Consideration of the design of devices is needed to accommodate impaired dexterity
Visual acuity	Necessary to operate specific devices	Prevalence of cataract, glaucoma, etc. in older adults with diabetes can impair ability to use devices	Visual acuity and ability to interact with device screens and buttons	Consider font size, screen visibility (e.g. high-contrast setting) and use of magnifying devices
Frailty and reduced activities of daily living	Limits self-management ability	Increased risk of frailty with poorer outcomes in those with micro-and macrovascular diabetes-related complications	FRAIL scale or Clinical Frailty Scale (CFS) [74]	Tailor device selection to fit older adult’s abilities and context
Social context and environment	Affects the feasibility of self-care	Different needs based on older adult’s living situation	Initial assessment of older adult’s living conditions and support system	Choice of device should consider social context (e.g. potential to share glucose information remotely with family members)

## Areas for future research and improvement

**Fig. 3:**
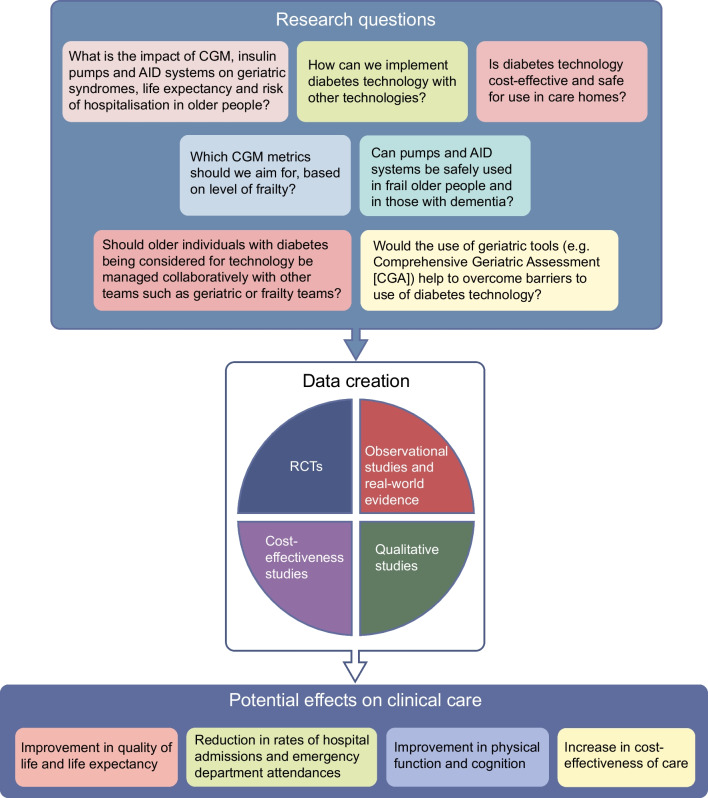
Research questions related to diabetes technology in older people. This figure is available as part of a downloadable slideset

The emerging body of evidence regarding the efficacy and safety of diabetes technology use among the older population is largely limited to individuals without comorbidities who are cognitively intact and living in their own homes. We have identified the following areas for future research and further development (Fig. 3):

- real-world observational studies and RCTs exploring the impact of CGM, pumps and AID on outcomes of interest to the ageing population (geriatric syndromes and life expectancy) and including individuals with a broad spectrum of frailty
- identification of strategies for implementing diabetes technology alongside other technology (blood pressure monitoring, cardiac monitoring, oxygen monitoring) to support frail older people at home and in long-term facilities, to avoid hospitalisations and readmissions
- research aimed at adapting diabetes technology to older people with diabetes, frailty and disability, for both people who manage independently and those needing assistance
- research on the efficacy, safety and cost-effectiveness of the use of diabetes technology in all living settings including care homes
- guidelines on the use of diabetes technology in older people with diabetes and cognitive and physical impairments
- recommendations on CGM-based metrics for older people, stratified by health status, comorbidities, frailty and life expectancy
- training on the use of technology for care homes staff looking after residents with diabetes
- interventions to improve e-literacy among older people with diabetes
- development of pumps and AID systems that can be operated remotely either by a caregiver or a relative.

We must also remain cognisant that most glucose sensors, and all current pumps, are worn externally; therefore, the available skin ‘real estate’ is a major factor in decisions around the use of technology. Ageing skin has high rates of dermatological conditions and may have less resilience to the adhesives/piercing required, which may limit its usage for many individuals. If people are on anti-platelet agents or anti-coagulants, bruising can be a major issue at insertion sites [77, 78]. Future research could focus on developing alternative adhesive materials or insertion techniques to address these challenges and improve the acceptance and usability of technology.

## Conclusions

Given the improvement in life expectancy of adults with diabetes, clinicians will soon face the challenge of providing care to more older people with long-duration type 1 diabetes or type 2 diabetes treated with insulin and co-existing long-term conditions. The heterogeneity characterising this group of individuals is a key factor in the pronounced glucose variability observed, as well as the increased risk of hypoglycaemia, effects of medical comorbidities and their ability to use diabetes technology. Individually tailored adoption of CGM devices, pumps and AID systems appears to be the most sensible strategy to improve TIR and reduce the risk of hypoglycaemia and hyperglycaemia in this group, which, in turn, could improve physical and cognitive function and quality of life; however, further well-designed research is needed to confirm these benefits.

While technology holds promise for enhancing the lives of older adults, it is important to be mindful of its limitations and potential pitfalls. Certain characteristics, such as advanced age, limited digital literacy, cognitive decline and physical impairments, should be considered when implementing technology solutions for older individuals. Additionally, factors such as privacy concerns, reluctance to adopt new technologies and the need for personalised support and training should also be considered when designing technology interventions for this population. By acknowledging these challenges and incorporating appropriate caveats, technology developers and healthcare professionals can ensure that technology is effectively used to support older adults while minimising potential risks and drawbacks.

Implementation of technology should be considered after a careful and comprehensive assessment of each individual, looking at multiple domains including social context and living setting. Training and education should be provided to family members and caregivers of frail older people with disability to support their use of diabetes technology.

## Supplementary Information

Below is the link to the electronic supplementary material.

Slideset of figures (PPTX 691 KB)

## Abbreviations

AID: Automated insulin delivery
CGM: Continuous glucose monitoring
SAP: Sensor-augmented pump
TBR: Time below range
TIR: Time in range
