# Supplementary material for: Ageing well with diabetes: the role of technology
Source: Diabetologia. 2024 Aug 13;67(10):2085–102. doi: 10.1007/s00125-024-06240-2 (PMC11446974; doi:10.1007/s00125-024-06240-2)
Supplement: Supplementary file 1 — Slideset of figures (PPTX 691 KB) [file 125_2024_6240_MOESM1_ESM.pptx]

## Slide 1
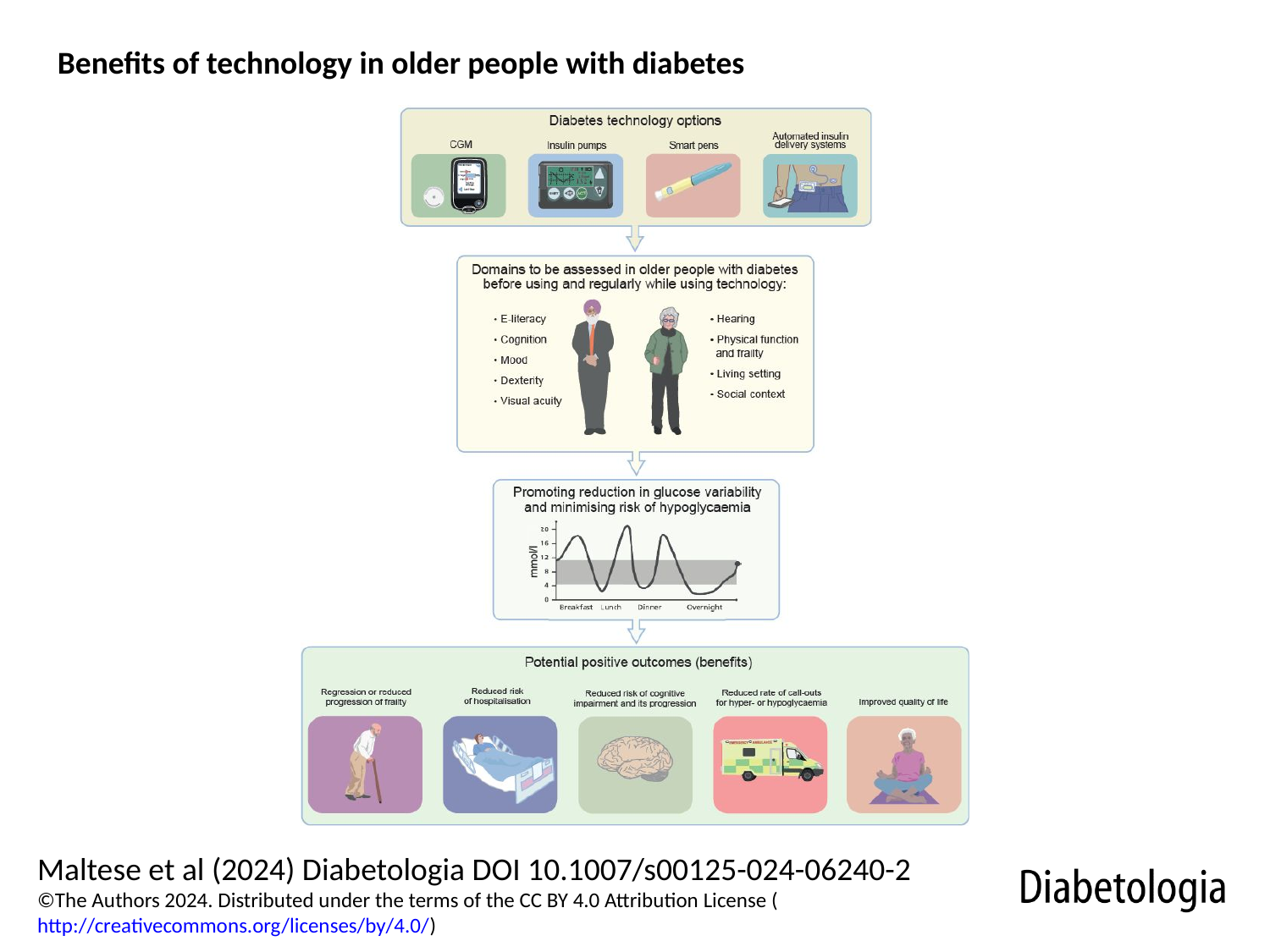

Benefits of technology in older people with diabetes
Maltese et al (2024) Diabetologia DOI 10.1007/s00125-024-06240-2
©The Authors 2024. Distributed under the terms of the CC BY 4.0 Attribution License (http://creativecommons.org/licenses/by/4.0/)

## Slide 2
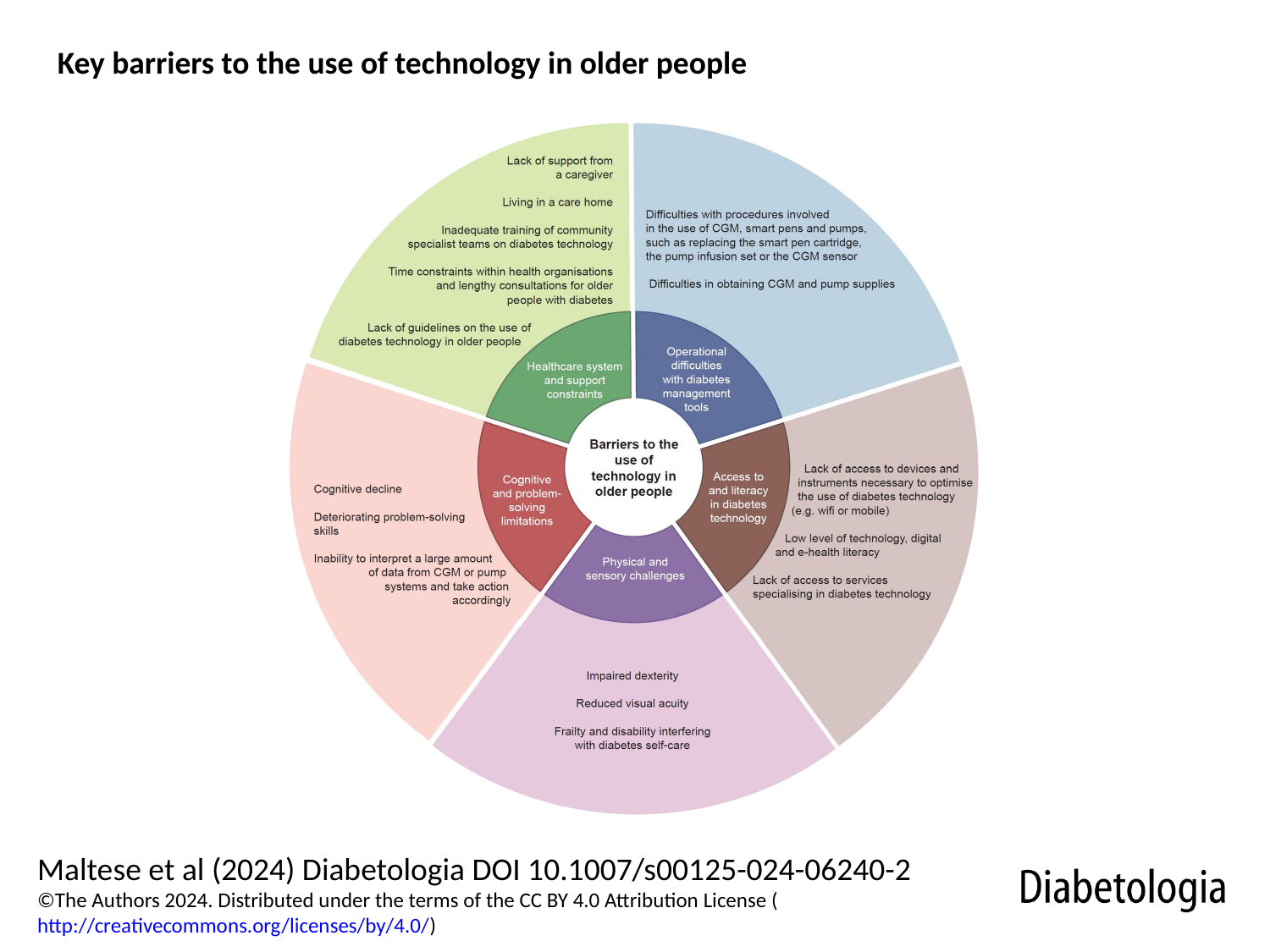

Key barriers to the use of technology in older people
Maltese et al (2024) Diabetologia DOI 10.1007/s00125-024-06240-2
©The Authors 2024. Distributed under the terms of the CC BY 4.0 Attribution License (http://creativecommons.org/licenses/by/4.0/)

## Slide 3
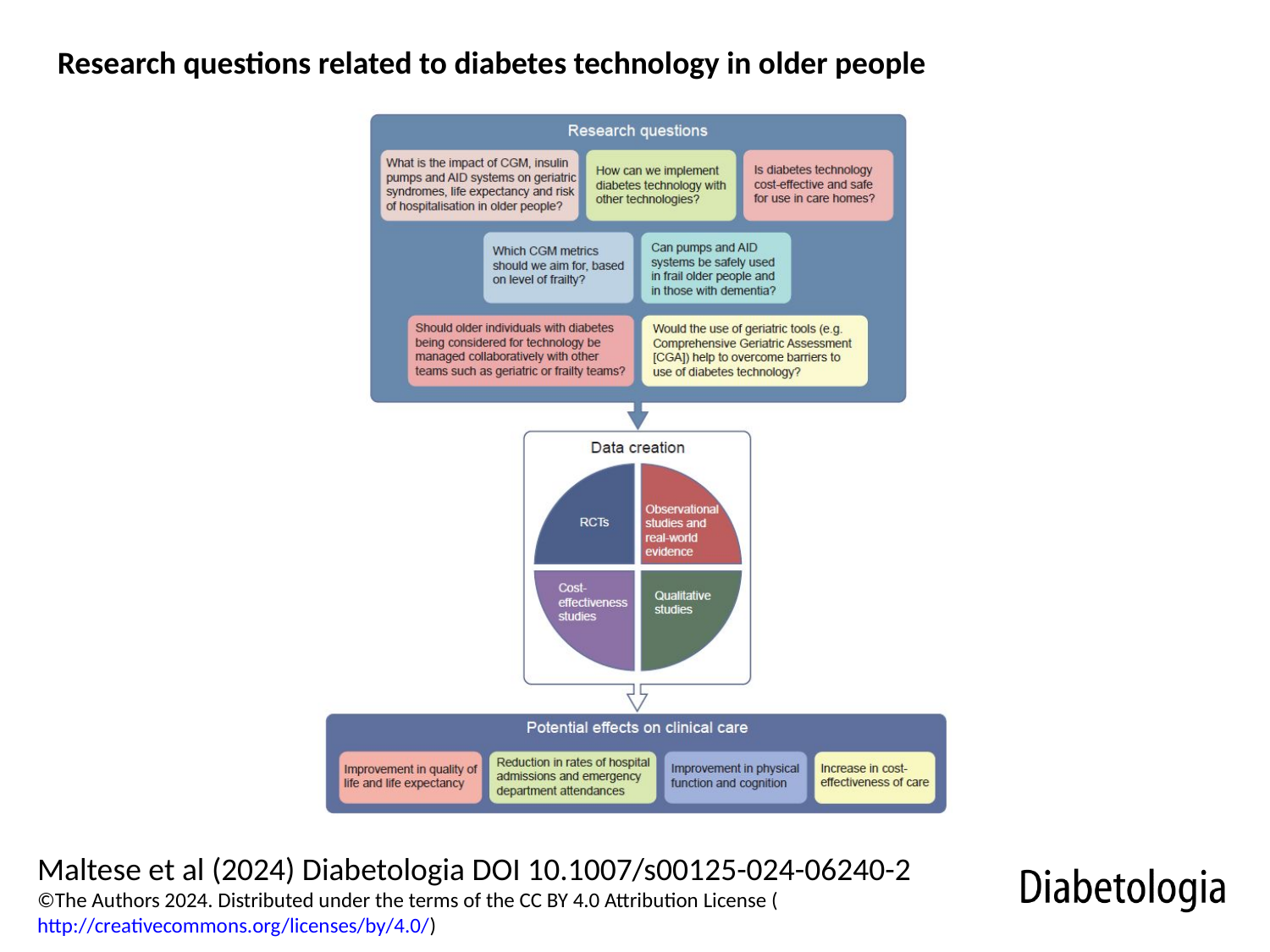

Research questions related to diabetes technology in older people
Maltese et al (2024) Diabetologia DOI 10.1007/s00125-024-06240-2
©The Authors 2024. Distributed under the terms of the CC BY 4.0 Attribution License (http://creativecommons.org/licenses/by/4.0/)
